# Special Issue “Molecular Research and Cellular Biology of Breast Cancer”

**DOI:** 10.3390/ijms27167081

**Published:** 2026-08-07

**Authors:** Andrea Nicolini

**Affiliations:** Department of Clinical and Experimental Medicine, Pisa University, 56126 Pisa, Italy; andrea.nicolini@med.unipi.it

Cancer is fundamentally a genetic and epigenetic disease [1,2], involving a complex landscape of interactions between cancer cells and other cells within the tumor microenvironment (TME). These interactions are sustained by intricate networks of molecular mechanisms and signaling pathways [3,4], which differ, at least in part, among cancer types and are commonly driven by genetic and epigenetic alterations in cancer cells. Overall, this constitutes the “specific” biology of cancer, which largely diverges from that of normal cells [5]. Thus, most expectations for improving our effectiveness in combating cancer rely on major advances in our understanding of cancer cell-specific biology. Accordingly, the initial part of this Special Issue (SI) includes five experimental research articles that shed light on molecular and genetic mechanisms underlying specific cancer biology, whereas the remaining articles, which have more direct clinical implications, focus on breast cancer prognosis and drug resistance.

The first research article, by Iozzo et al. [6], deals with the temporal heterogeneity of hormone receptors and HER2 in breast cancer cutaneous metastases compared with the primary tumor. Cutaneous metastases are uncommon in cancer patients, ranging from 0% to 10% when all cancer types are considered [7,8], and from approximately 2% to 5% in breast cancer [9]. In this study, the authors retrospectively examined ER, PgR, and HER2 status in 28 paired primary tumors and cutaneous metastatic lesions selected from a cohort of 669 breast cancer patients treated at a tertiary care centre. ER and PgR expression were evaluated as the percentage of tumor cell nuclei showing unequivocal specific staining, and a cut-off of ≥1% defined receptor positivity. HER2 status was assessed by IHC and scored as 0, 1+, 2+, or 3+ following ASCO/CAP recommendations. HER2-low status was defined as tumors showing IHC 1+ staining or IHC 2+ staining without evidence of HER2 gene amplification. Most ER- and PgR-negative cases in the primary tumor remained unchanged in cutaneous metastases, whereas ER- and PgR-positive cases in the primary tumor became negative in cutaneous metastases at rates ranging from 23% to 25%. Furthermore, in 61% of patients with HER2-0 status in the primary tumor, HER2 was classified as HER2-low in cutaneous metastases, whereas 25% of patients with HER2-positive primary tumors were classified as having HER2-0 cutaneous metastases. Underscoring the specificity of cancer cell biology, the authors conclude that discordance in these receptors “reflects dynamic tumor evolution” (Figure 1).

Cellular clusters expressing germline-associated genes constitute the main population presenting cancer-testis antigens (CTAs). Recently, the active expression of specific cancer-testis genes has been confirmed [10], and the potential role of CTAs, which are normally confined to the testis during postnatal life, in breast cancer evolution and prognosis has been elucidated [11,12,13]. Accordingly, the subsequent study by Khodayari et al. [14], based on published datasets, identifies and investigates cellular populations in breast cancer that express genes associated with male germ cell development. Expression of the identified genes was experimentally validated using quantitative RT-PCR in 28 breast tumor samples. Tumor heterogeneity and cellular clustering were also examined using single-cell RNA sequencing (scRNA-seq). Overall, 455 overexpressed genes related to fetal primordial germ cells (PGCs), particularly those of the male gonad, were identified. Significant overexpression of *CCNB1*, *CCNB2*, *PTTG1*, *RACGAP1*, and *UBE2C* was observed in the 28 tumor samples, whereas scRNA-seq analysis revealed 14 distinct cell clusters. Breast tumor stromal cells represented the principal source of germline-associated gene expression.

Atypical Protein Kinase C (aPKC) comprises a family of serine/threonine kinase isoforms [15] that regulate several crucial signaling pathways in cancer [15,16]. Overexpression of PKC iota (PKC-ι) has been reported in numerous cancer types, including breast cancer [17,18,19]. The study by Oishee et al. [20] focuses on the role of PKC-ι, a bona fide human oncogene. In this research, ICA-1S, a PKC-ι-specific inhibitor, significantly inhibited cell growth and promoted apoptosis in both BT-549 and MCF-7 breast cancer cell lines. Treatment with ICA-1S and small interfering RNA (siRNA) also reduced the expression levels of c-Jun, a component of the MAPK/JNK pathway, and TNF-α in both cell lines. PKC-ι was strongly associated with c-Jun, through which it regulated the MAPK/JNK pathway. Moreover, ICA-1S induced c-Jun degradation and reduced c-Jun mRNA levels. The authors conclude that PKC-ι plays a crucial role in regulating breast cancer progression and represents a potential therapeutic target.

Resistance to endocrine therapy in estrogen receptor-positive (ER+) tumors [21] and variable responses to anti-HER2 therapy in HER2+ tumors [22] are two major challenges in the management of breast cancer. The research article by Ji et al. [23] describes claudin-8 (CLDN8), a tight junction protein, as a potential prognostic biomarker and predictor of therapeutic response in breast cancer. In this study, the association between CLDN8 gene expression and clinical outcomes, as well as response to therapy, was examined in breast cancer patient cohorts. The effects of altered CLDN8 expression on cell behaviour and drug sensitivity were also investigated in breast cancer cell models. Elevated CLDN8 expression was significantly associated with increased disease-free survival (DFS) among estrogen receptor-negative (ER−) patients. In particular, tumors with CLDN8 overexpression were associated with better outcomes in patients treated with surgery alone or endocrine therapy, whereas those receiving chemotherapy (including neoadjuvant chemotherapy) or anti-HER2 therapy paradoxically exhibited poorer survival and greater therapy resistance. In vitro, CLDN8 knockdown reduced sensitivity to endocrine therapies, HER2-targeted agents, and chemotherapeutic drugs, reflecting the observed clinical patterns. Overall, the authors suggest that CLDN8 expression may be useful in guiding personalised therapy and may represent a candidate target for overcoming treatment resistance in breast cancer.

The final paper, authored by Mull et al. [24], begins by noting that adenosine triphosphate (ATP) concentrations are markedly elevated in the TME compared with normal tissue and that P2Y2 is an ATP-activated purinergic receptor involved in cellular signaling across multiple tissues. Activated by extracellular ATP, P2 receptors regulate numerous biological functions [25] and are classified into two families: P2X, ligand-gated ionotropic channels; and P2Y, metabotropic G-protein-coupled receptors (GPCRs). The study focuses on the latter [25]. The authors underscore that “purinergic signaling, specifically the P2Y2 receptor, has been understudied in breast cancer and remains an intriguing target to further investigate therapy” [26,27,28]. In this research, rapid phenotypic responses occurring within seconds to minutes after ATP-mediated purinergic receptor activation, and how these responses may differ between breast cancer and non-tumorigenic cells, were investigated. ATP-activated P2Y2 signaling induced substantially greater increases in intracellular Ca2+ levels in non-tumorigenic breast epithelial cells than in their tumorigenic and metastatic counterparts. In non-tumorigenic cells, unlike metastatic cells, this response was followed by actin polymerisation and redistribution to the cell periphery. Furthermore, in non-tumorigenic cells, following ATP stimulation, a P2Y2 antagonist reduced intracellular Ca2+ levels to baseline values, counteracted actin remodelling, and importantly, increased cell dissemination from spheroids. The authors conclude that the absence of Ca2+ signaling and actin remodelling in metastatic breast cancer cells is attributable to reduced P2Y2 expression, which correlates with poorer overall survival in breast cancer and likely represents an adaptive mechanism for evading cellular responses to extracellular ATP.

Two additional articles are included in this Special Issue. The first, by Stanciu et al. [29], examines the prognostic role of demographic, clinical, and tumor-related characteristics in determining the efficacy of cyclin-dependent kinase 4 and 6 (CDK4/6) inhibitors, whereas the second article, by Faur et al. [30], investigates the ability of peripheral blood parameters to predict responses to neoadjuvant chemotherapy. It is well established that patient characteristics, including age, ECOG performance status, previous treatments, and comorbidities, can significantly affect progression-free survival (PFS) and overall survival (OS) in ER+ HER2− breast cancer patients treated with endocrine therapy (ET) and/or CDK4/6 inhibitors [31,32,33]. Accordingly, the former study evaluated patient demographics, tumor characteristics, treatment regimens, PFS, OS, time on treatment (TOT), and the influence of clinical and biological factors in 95 patients with metastatic ER+ HER2− breast cancer treated with CDK4/6 inhibitors (ribociclib, palbociclib, or abemaciclib) in combination with endocrine therapy. Greater tumor aggressiveness and poorer survival were observed in younger patients (<50 years). Ribociclib was associated with the greatest survival benefit, particularly among younger patients, whereas older patients (>50 years) exhibited higher rates of comorbidities and treatment-related toxicity. The authors conclude that these findings underscore the importance of personalised treatment strategies based on patient age, comorbidities, and tumor biology.

Within the breast cancer TME, tumor-infiltrating lymphocytes (TILs) are strongly associated with pathological complete response (pCR) following neoadjuvant chemotherapy (NACT) [34,35,36]. In contrast, certain TIL subtypes, such as regulatory T lymphocytes (Tregs) and myeloid-derived suppressor cells (MDSCs), may reduce the effectiveness of NACT [37]. However, few studies have examined the relationship between the peripheral immune system and NACT response in breast cancer [38,39]. Therefore, the latter study investigated the usefulness of age, TNM stage, histological type, molecular subtype, platelet-to-lymphocyte ratio (PLR), neutrophil-to-lymphocyte ratio (NLR), lymphocyte-to-monocyte ratio (LMR), systemic immune-inflammatory index (SII), and prognostic nutritional index (PNI) in predicting tumor response to NACT. The analysis revealed a statistically significant association between pCR and NLR in patients with values between 1 and 3. The optimal cut-off values for PLR and LMR were 120.45 and 12.34, respectively, and 80% and 78% of patients with values below these thresholds achieved pCR. Lower pre-treatment values of NLR, PLR, SII, and LMR were significantly associated with pCR compared with the non-pCR subgroup. The authors conclude that, in breast cancer patients undergoing NACT, NLR, PLR, SII, and LMR, unlike PNI, are predictive biomarkers of pCR and may help guide personalised treatment strategies.

Goidescu et al. [40] were inspired by the observation that “multigene panel testing for hereditary breast and ovarian cancer is becoming a standard in medical care” [41,42]. The study focuses on moderate- and low-risk breast cancer gene expression in a Romanian population and reports on 255 consecutive breast cancer cases that met the criteria for genetic testing. In 98 of them, 104 pathogenic variants (PVs) were identified by next-generation sequencing: 69% were in high-risk genes, 20% in moderate-risk genes, 3% in low-risk genes, and 8% in genes for which evidence remains insufficient. The authors state that their study provides a valuable contribution toward a more comprehensive understanding of the genetic factors influencing cancer development, improving screening protocols, and refining risk models for patient management. The last four papers, comprising two original research articles and two reviews, focus on mechanisms of drug resistance. Specifically, the research article by Dlugosz-Pokorska et al. [43], entitled “Synergistic Effects of Oxaliplatin, 5-Fluorouracil, and Novel Synthetic Uracil Analog U-359 on Breast Cancer Cell Carcinogenesis”, examines the cytotoxic effects of U-359, oxaliplatin (Ox), and 5-fluorouracil (5-FU) in MCF-7 and MCF-10A cells using MTT and RealTime-GLO assays. The results demonstrated that U-359, in combination with Ox and 5-FU, increased cytotoxicity compared with the individual treatments. The Bliss Independence Model revealed strong synergistic interactions between U-359 and either Ox or 5-FU, particularly with respect to reducing ABCB1 and NF-κB expression levels. The authors conclude that U-359, when combined with Ox and 5-FU, may overcome chemotherapy resistance in breast cancer by enhancing apoptosis and modulating mechanisms of drug resistance.

The research article by Rojas-Salazar et al. [44] investigates therapeutic strategies aimed at overcoming drug resistance and improving outcomes in HER2+ breast cancer. In these patients, amplification or overexpression of the ERBB2 (HER2) gene is commonly associated with aggressive tumors and poor prognosis, while drug resistance frequently develops following single-agent therapies targeting the ERBB2 pathway [45,46]. The study explored potential drug synergies by analysing gene–drug relationships and combination therapies directed against the ERBB2 pathway. Using gene co-expression network analysis, the authors identified 23 metabolic pathways exhibiting significant cross-linking of gene interactions, including pathways involving EGFR tyrosine kinase inhibitors, PI3K, mTOR, and others. Combinations of cetuximab with either lapatinib or erlotinib, as well as the combination of erlotinib and lapatinib, were predicted to exert synergistic effects and therefore to enhance therapeutic efficacy through the simultaneous targeting of multiple genes and signaling pathways.

The first of the two reviews, authored by Postigo-Corrales et al. [47], focuses on the molecular mechanisms underlying intrinsic and acquired resistance to docetaxel in breast cancer. Major resistance mechanisms include alterations in drug targets (microtubules), increased drug efflux, suppression of apoptosis, activation of survival signaling pathways, epithelial-to-mesenchymal transition (EMT), and enrichment of cancer stem cells. Therapeutic interventions targeting the tumor microenvironment (TME), exploiting immunotherapy, and implementing adaptive dosing schedules have been proposed as strategies to restore docetaxel sensitivity in preclinical studies [48]. At the same time, clinical approaches, including personalised treatment regimens, have been explored to delay or prevent the development of docetaxel resistance in breast cancer [49,50,51]. The review underscores the multifactorial nature of docetaxel resistance, which consequently requires multiple therapeutic countermeasures. The authors highlight that identifying predictive biomarkers, developing targeted agents capable of reversing resistance, and designing rational combination strategies to improve patient outcomes remain major objectives of ongoing research in this field.

The review by Ferrari et al. [52] originates from the observation that a large proportion of patients with advanced breast cancer present with ER+ HER2− disease and that the recent combination of endocrine therapy (ET) with CDK4/6 inhibitors has significantly improved clinical outcomes. However, intrinsic and acquired endocrine resistance remain major unresolved challenges. Consequently, this review, which examines the principal mechanisms underlying resistance to ET alone or in combination with biological agents, as well as novel therapeutic strategies to overcome such resistance, is of considerable scientific interest.

The review initially addresses the major mechanisms of endocrine resistance, including genetic aberrations (mutations, fusions, and amplifications) affecting the ligand-binding domain of the ESR1 gene, alterations involving regulators of the ER pathway and other signaling cascades, metabolic reprogramming, and additional cellular processes. Mechanisms responsible for resistance to ET and/or CDK4/6 inhibitors are subsequently grouped and discussed separately. These include genetic aberrations affecting cell-cycle regulation, activation of alternative molecular pathways, and alterations in transcriptional and epigenetic regulators. Additional mechanisms potentially involved include acquired CDK6 amplification, alterations in the c-Myc oncogene, immunological changes within the TME, and sustained cellular proliferation despite CDK inhibition.

The review then focuses on therapeutic strategies commonly recommended following disease progression during treatment with endocrine therapy plus CDK4/6 inhibitors. Depending on whether ER-dependent signaling is maintained or lost, different therapeutic options are available. Antibody-drug conjugates (ADCs), consisting of antibodies directed against tumor antigens linked to cytotoxic agents, represent another recently introduced therapeutic class. Trastuzumab deruxtecan and sacituzumab govitecan are two representative ADCs currently available for clinical use.

Complete estrogen receptor antagonists (CERANs), selective estrogen receptor modulators (SERMs), SERM/SERD hybrids (SSHs), selective estrogen receptor covalent antagonists (SERCAs), selective human ER partial agonists (ShERPAs), PROteolysis TArgeting Chimeras (PROTACs), and selective androgen receptor modulators (SARMs) constitute the next generation of endocrine agents, most of which are currently under clinical development.

Regarding immunotherapy, in addition to treatment with macrophage inhibitors and ESR1mut vaccines, the authors describe a therapeutic protocol they have developed. This protocol is based on the synergistic activity of a beta-interferon/iterleukin-2 sequence administered in combination with conventional endocrine therapy in patients with relapsed ER+ endocrine-dependent breast cancer. After discussing preliminary findings from a pilot study, reported on several occasions [53,54,55] (Figure 2), the authors present the final results of a subsequent retrospective study recently published [56,57], in which 95 controls were compared with 42 treated cases. Median PFS and OS were significantly prolonged in the 42 treated patients compared with the 95 controls (Figure 2).

The authors hypothesized that “in responsive metastatic disease a stable or decreased tumor burden and a lower genetic instability due to the quiescent state (G0–G1 state) of tumor cells induced by anti-estrogens is also likely to reduce the immune evasion and immune inhibition. This favours the immune attack stimulated by the concomitant immune therapy” [43]. The authors further comment that “this mechanistic rationale is different from that of CDK4/6is; therefore, the proposed investigational immunotherapy can be a reasonable alternative for patients who maintain ER signaling after they have progressed with ET and CDK4/6is”. They conclude by stating that “better patient selection for different therapeutic approaches is expected through the identification of more precise biological and genetic markers”. In particular, they underscore that “liquid biopsy may provide a real-time portrait of the disease, offering insights into mechanisms driving ET resistance and cancer progression”.

## Figures and Tables

**Figure 1:**
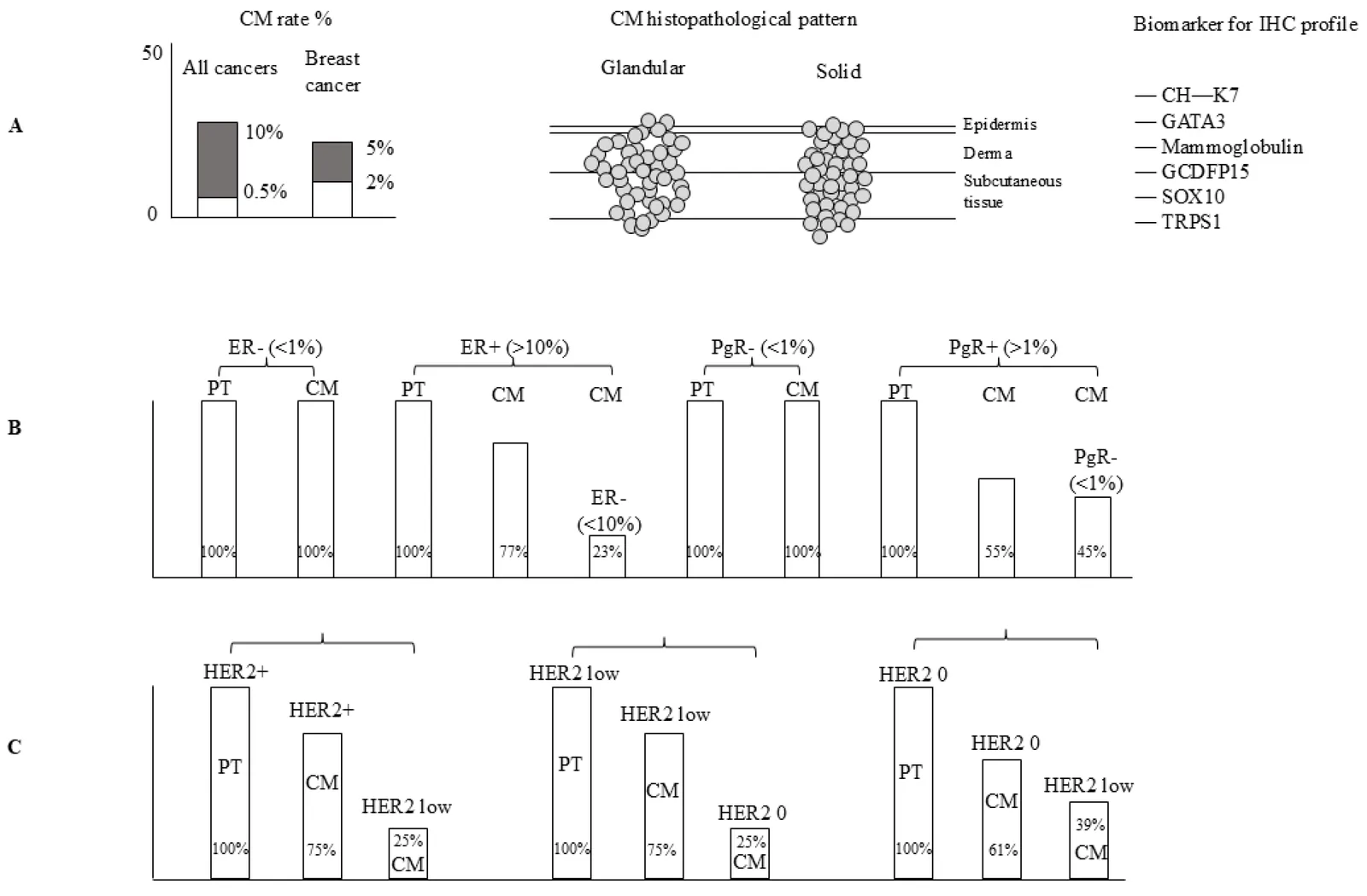
(**A**) CM rate in all different cancer types and in breast cancer; histopathological pattern of CM and biomarkers commonly used to define the IHC profile; (**B**) ER and PgR discordance rate; (**C**) HER2 discordance rate. CM: cutaneous metastases; IHC: immunohistochemistry; PT: primary tumor; reproduced from ref. [6].

**Figure 2:**
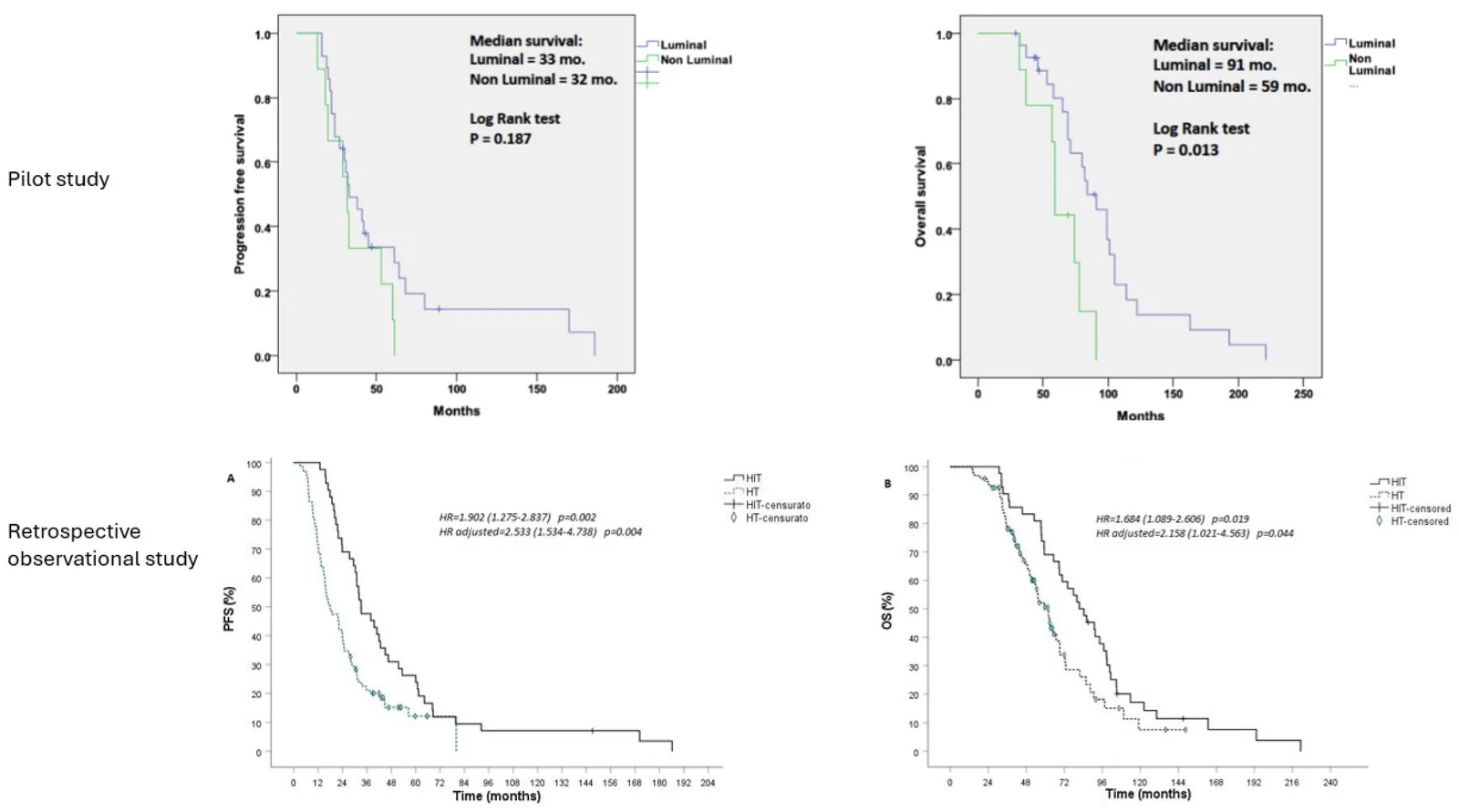
Upper half, left side: Progression free survival from hormone therapy in 37 endocrine-dependent metastatic breast cancer patients (by the Kaplan–Meier method); median survival time and 95% CI: Luminal (n = 28) 33, 20.6–45.4 months; non-Luminal (n = 9) 32, 23.2–40.7 months. Upper half, right side: Overall survival from hormone therapy in 37 endocrine-dependent metastatic breast cancer patients (by the Kaplan–Meier method); median survival time and 95% CI: Luminal (n = 28) 91 months (95% CI: 73.8–108.2); non-Luminal (n = 9) 59 months (95% CI: 56.1–61.9). Reprinted from Curr Med Chem, Nicolini A, Barak V, Biava P, Ferrari P, Rossi G, Carpi A. The use of immune therapy to treat metastatic breast cancer; pages 941–962, copyright 2026, with permission from Bentham. Lower half: Progression-free survival correlated with hormonal therapy (survival median time of HIT 33 months (95%CI: 24–42); survival median time of HT 18 months (95%CI: 12–23)) (A). Overall survival correlated with hormonal therapy [survival median time of HIT 81 months (95%CI: 64–99); survival median time of HT 62 months (95%CI: 54–70)] (B); reproduced from ref. [46].
